# Genetic Improvement of Sawn-Board Stiffness and Strength in Scots Pine (*Pinus sylvestris* L.)

**DOI:** 10.3390/s20041129

**Published:** 2020-02-19

**Authors:** Irena Fundova, Henrik R. Hallingbäck, Gunnar Jansson, Harry X. Wu

**Affiliations:** 1 Umeå Plant Science Centre, Department of Forest Genetics and Plant Physiology, Swedish University of Agricultural Sciences, 90183 Umeå, Sweden; henrik.hallingback@slu.se; 2 Skogforsk (Forestry Research Institute of Sweden), 91821 Sävar, Sweden; 3 Skogforsk (Forestry Research Institute of Sweden), 75183 Uppsala, Sweden; gunnar.jansson@skogforsk.se; 4 Beijing Advanced Innovation Centre for Tree Breeding by Molecular Design, Beijing Forestry University, Beijing 100083, China; 5 CSIRO National Research Collection Australia, Black Mountain Laboratory, Canberra ACT 2601, Australia

**Keywords:** Structural timber, non-destructive testing, wood quality, modulus of elasticity, modulus of rupture, acoustic velocity, heritability, genetic correlation, tree breeding, genetic improvement

## Abstract

Given an overall aim of improving Scots pine structural wood quality by selective tree breeding, we investigated the potential of non-destructive acoustic sensing tools to accurately predict wood stiffness (modulus of elasticity, MOE) and strength (modulus of rupture, MOR) of sawn boards. Non-destructive measurements of wood density (DEN), acoustic velocity (VEL) and MOE were carried out at different stages of wood processing chain (standing trees, felled logs and sawn boards), whilst destructively measured stiffness and strength served as benchmark traits. All acoustic based MOE and VEL estimates proved to be good proxies (*r*_A_ > 0.65) for sawn-board stiffness while MOE_TREE_, VEL_HIT_ and resistograph wood density (DEN_RES_) measured on standing trees and MOE_LOG_ and VEL_FAK_ measured on felled logs well reflected board strength. Individual-tree narrow-sense heritability ($h_{i}^{2}$) for VEL, MOE and MOR were weak (0.05–0.26) but were substantially stronger for wood density (0.34–0.40). Moreover, additive genetic coefficients of variation for MOE and MOR were in the range from 5.4% to 9.1%, offering potential targets for exploitation by selective breeding. Consequently, selective breeding based on MOE_TREE_, DEN_RES_ or stem straightness (STR) could improve several structural wood traits simultaneously.

## 1. Introduction

Wood stiffness and strength are important structural-timber traits that determine the suitability of wood for construction purposes. Stiffness and strength, expressed as modulus of elasticity (MOE) and modulus of rupture (MOR), respectively, refer to an amount of load that an object can resist without deformation and the stress needed to cause a failure [1]. Many forest tree breeding programs, including that of Scots pine (*Pinus sylvestris* L.), have however been prioritizing mainly stem volume improvement whilst wood quality traits such as stiffness and strength have been ignored. This approach will create a potential problem for species whose stem volume is negatively correlated with wood quality traits [2,3,4,5,6]. Furthermore, long rotation, typical for northern latitudes, makes forest tree improvement complicated because it is not feasible to postpone selection until trees mature. Some traits are not even expressed until the wood is processed and it is therefore necessary to seek traits that can be measured non-destructively on standing trees in early stages of the rotation period and that can, at the same time, provide a reliable image of the final-products’ properties.

Only a limited number of studies dealing with structural timber traits measured on sawn boards have been conducted at the genetic level until now. Directly measured wood stiffness was studied e.g., in radiata pine (*Pinus radiata* D. Don) [7], Douglas-fir (*Pseudotsuga menziesii* (Mirbel) Franco) [8], hybrid larch (*Larix* spp.) [9] or *Eucalyptus nitens* [10], whereas the potential for genetic improvement of sawn boards’ shape stability was explored in Scots pine [11] and Norway spruce (*Picea abies* (L.) Karst) [12]. Such studies require a large number of trees with known pedigree, arranged following a properly designed field test, to be harvested. Consequently, the harvested trees do not contribute to the field test anymore. In Sweden, systematic forest tree breeding was launched in 1950s [13] and, due to the long rotation time, there are no well-designed Scots pine progeny field tests available yet that would have reached rotation age. Fortunately, Scots pine trees in Sweden often reach the minimum sawmillable dimensions at around half the rotation period and are therefore accessible as a result of commercial thinning operations.

Direct measurements of stiffness and strength require destructive bending stress testing that lead to sample destruction and thus are inappropriate from the perspective of tree breeding as well as practical utilization. However, acoustic sensing technology offers a suitable non-destructive proxy for wood stiffness. Dynamic modulus of elasticity, calculated as squared acoustic velocity multiplied by wood density, represents an indirect measure of stiffness [14]. The acoustic velocity can be derived from 1) acoustic resonance (AR) or 2) time of flight (TOF). The AR approach is more suitable for felled logs and sawn boards, as it requires cut ends that serve as acoustic wave reflectors: longitudinal stress waves, generated by a hammer tap, reverberate within a log or board. The TOF approach is, on the other hand, applicable on standing trees, as it measures time of flight of a stress wave between two probes imbedded into a stem. The AR approach is considered to be more accurate than TOF [15] because the effective propagation distance is only ~1 m for TOF, whilst it is several times the length of a log/board for AR as the sound travels forth and back between the cut ends. Furthermore, the TOF measurement is restricted only to a narrow column of outerwood that comprises of a limited number of annual rings, whilst AR considers a whole log/board’s profile [16,17,18]. Nevertheless, strong correlations between acoustic velocities estimated using AR and TOF approaches have been reported by a number of studies [19,20,21,22].

The aim of this study was to: (1) compare the benchmark stiffness obtained from destructive testing with that assessed non-destructively at different stages of the wood processing chain, namely on standing trees, felled logs and sawn boards; (2) estimate the genetic and phenotypic variation and heritability of all structural timber traits and calculate phenotypic and additive genetic correlations among them, as well as with wood density and growth traits; and (3) estimate the extent to which structural sawn timber quality traits may be improved by selective breeding.

## 2. Materials and Methods

### 2.1. Test Material

A Scots pine full-sib progeny test “Älvkarleby” located in central Sweden (#S22F791110E, 60°32′35” N, 17°26′12” E, 25 m a.s.l.) was chosen for this project because a systematic thinning, scheduled for this site, offered a unique opportunity to conduct a sawmill study. The test was established by Skogforsk in 1979 using 90 full-sib families generated by 24 parents according to partial diallel mating design [23] plus five commercial checklots. The parents were plus-trees phenotypically selected in diverse forest stands throughout central Sweden (the latitudes and longitudes of origin ranged from 59°58′ N to 62°04′ N and from 12°54′ E to 16°42′ E, respectively). Scions from these plus trees were grafted on root stocks and used for establishing a clonal seed orchard, in 1958 (#S22FP1462 “Forn-Wij” 60°54′ N, 16°42′ E), in which the controlled crosses were later performed. Progenies generated from these crosses were subsequently planted on Älvkarleby test site as one-year-old seedlings with 2 × 2 m spacing using a completely randomized block design. The test originally included eight blocks but one of them suffered from excessive mortality and was no longer maintained. Consequently, only seven blocks were included in the study. The soil was a podzol with a 3–6 cm humus layer.

### 2.2. Standing Trees

All trees were first scored for vitality using a 4-point scale. Live trees (1896) were then measured for diameter at breast height (1.3 m, DBH) and assessed for stem straightness (STR) using a 9-point scale (9 = completely straight). Wood density (DEN_RES_) was measured on each tree in south-north direction at the height of ca 1.2 m above ground using micro-drill Resistograph IML-RESI PD300 (Instrumenta Mechanic Labor, Germany). Drilling profiles were adjusted according to [24] in order to eliminate an increasing trend caused by needle friction and to remove bark. Wood density was calculated as a mean value of the adjusted profiles divided by four for better scaling. Hitman ST300 (Fiber-gen, New Zealand) was used to measure standing-tree acoustic velocity (VEL_HIT_). Its two probes were hammered into the southern part of each stem ca 90 cm apart and two groups of eight consecutive readings were averaged for calculating the dynamic modulus of elasticity (MOE_TREE_) as

(1) $${\mathrm{MOE}}_{\mathrm{TREE}}={\mathrm{VEL}}_{\mathrm{HIT}}^{2}\cdot {\mathrm{DEN}}_{\mathrm{RES}}.$$

All wood quality measurements were taken with an effort to avoid branches, knots and compression wood. Trees were measured during the same season (August 2017) and variation in their moisture content was considered negligible (no adjustments were made). 

### 2.3. Logs

The progeny test was systematically thinned in December 2017 (after 38 years in the field) by harvesting every third diagonal row running from southeast to northwest. In addition, every 11th–12th row running from north to south was harvested in order to create strip-roads suitable for log transportation. Among the trees thus harvested, a subset of 496 trees having the best vitality scores, being without any major damages or defects below the height of 4 m (due to e.g., major stem breakage, multiple stems, major ramicorns, rot) and showing a DBH larger than 15 cm was selected for the sawmill study. Bottom, 3.3 m long sawlogs from selected trees were carefully marked and transported to sawmill “Gösta Färdigh Sågverks AB” in Kalvsvik, Sweden. Prior to the actual sawing, the exact length (*L*) of all logs was recorded. In May 2018, acoustic resonance, induced by an external hammer, was measured using an Android application Resonance Log Grader (Fakopp Enterprise Bt., Hungary). Dynamic modulus of elasticity for logs (MOE_LOG_) was calculated as

(2) $${\mathrm{MOE}}_{\mathrm{LOG}}={\mathrm{VEL}}_{\mathrm{FAK}}^{2}\cdot {\mathrm{DEN}}_{\mathrm{RES}}$$

where DEN_RES_ is resistograph density measured on standing trees and VEL_FAK_ is acoustic velocity calculated from frequency (*f*_FAK_) of the first vibration mode, as

(3) $${\mathrm{VEL}}_{\mathrm{FAK}}=2\cdot L\cdot f_{FAK}.$$

### 2.4. Boards

The logs were sawn though the pith and edged using circular saws gaining two 50 × 100 mm boards per log (marked A and B). In total, 992 sawn boards were stored in two piles according to the group assigned, loaded with an extra pile of boards on top, covered with a portable roof, and left to air-dry over summer. 

#### 2.4.1. Non-Destructive Assessment of Wood Stiffness and Density

In autumn, acoustic resonance, moisture content, weight and wane were measured on all air-dried boards. The MTG Timber Grader (Brookhuis MicroElectronics), approved as a grading tool [25], was used to measure acoustic resonance on sawn boards. The boards were placed on two supports, three meters apart, and the resonance frequency of an impulse, induced by an integrated electric hammer, was recorded for each board. Modulus of elasticity was calculated as

(4) $${\mathrm{MOE}}_{\mathrm{BOARD}}={\mathrm{VEL}}_{\mathrm{MTG}}^{2}\cdot {\mathrm{DEN}}_{\mathrm{VOL}}$$

where DEN_VOL_ is volumetric mass density of a board estimated as a mass over volume and VEL_MTG_ is acoustic velocity calculated as

(5) $${\mathrm{VEL}}_{\mathrm{MTG}}=2\cdot L\cdot f_{MTG}$$

where *f*_MTG_ is resonance frequency at the first mode of vibration and *L* is length of a board corresponding to the length of a log. At this point, maximum wane depth was recorded and the moisture content (MC) of each board (mean at 15.3%) was measured by applying a two-pin moisture meter (Delmhorst RDM-2S) lengthwise 0.5 m from to the top end of the outer face of the board [26]. The variables VEL_MTG_, MOE_BOARD_ and DEN_VOL_ were thereafter adjusted to the standard moisture content of 12% with the aid of a simple linear regression. As these variables were measured on both boards (A and B) from each log, average values of the two measurements were used in statistical analyses.

#### 2.4.2. Fibre Orientation Scanning

**Figure 1:**
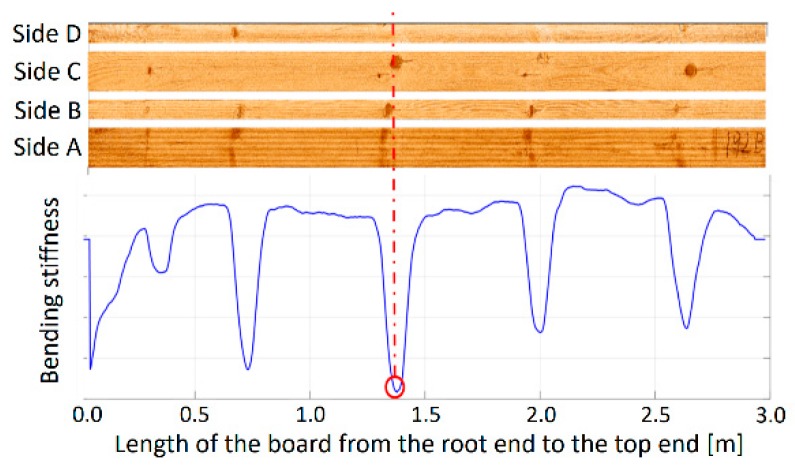
Prediction of critical selections.

Subsequently, the B-pile boards were shortened to the same length of 3 m and planed on the outer face side and on both long edges, reducing the dimension to 47 × 95 mm, in order to facilitate the scanning of fibre orientation. The outer face and long edges of the planed boards were then scanned by a WoodEye scanner (WoodEye AB, Sweden) located at Linnaeus University in Växjö, Sweden. The WoodEye scanner, equipped with four sets of cameras and lasers, utilizes a so-called “tracheid effect”, i.e., detects irregularities in wood based on differences in light scattering. In this study, information about the weakest points detected from image analysis [27] were used in subsequent destructive testing (Figure 1).

#### 2.4.3. Destructive Measurements of Wood Stiffness and Strength

Finally, the B-pile set of boards (496) was subjected to a destructive four-point bending test at the Asa Experimental Forest and Research Station belonging to the Swedish University of Agricultural Sciences in order to measure stiffness (static modulus of elasticity, MOE_S_) and strength (modulus of rupture, MOR). Before the testing, boards were measured for width and thickness at three positions along one of the long edges of each board. Also, MC was again recorded using the same moisture meter as mentioned above (mean at 15.2%), but this time at the three positions on the board mentioned previously. Average values of the three measurements were used for further analyses and adjustments.

**Figure 2:**
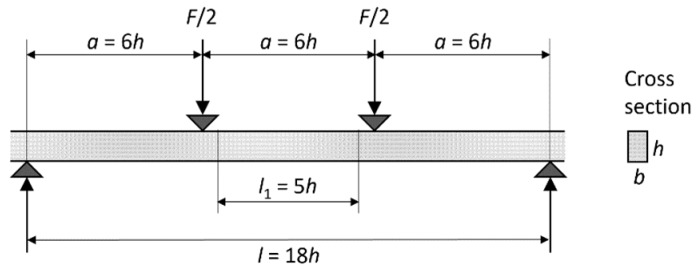
Schema of four-point bending test according to EN408, where *F* is load increment, *a* is distance between load points, *l*_1_ is central gauge length, *l* is test span, *h* is board width and *b* is board thickness.

**Figure 3:**
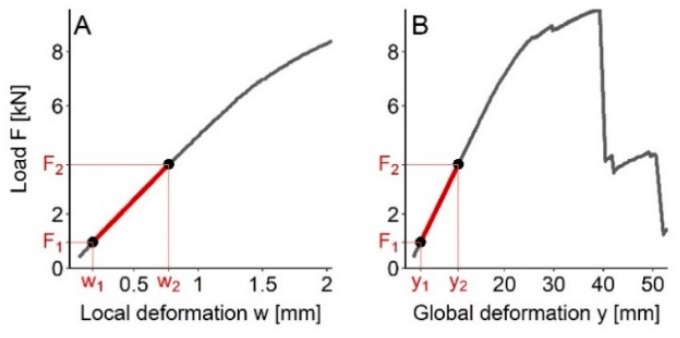
Load-deformation curves showing values used for estimation of local (**A**) and global (**B**) modulus of elasticity. *F*_1_ and *F*_2_ represent applied load calculated as 10% and 40% of the maximum load, respectively, and *w*_i_ and *y*_i_ are local and global deformations corresponding to *F*_i_, respectively.

The destructive testing was performed according to the EN 408 standard (Figure 2) [28]. A four-point bending test was applied in the way that the weakest point detected by WoodEye was placed in the center. The weakest points located less than 75 cm from either end were however not considered because the measurements would not be practically feasible. Local (MOE_S.local_) and global (MOE_S.global_) moduli of elasticity were estimated according to Equations (6) and (7), respectively. The former represents mid-span deflection, whilst the latter provides total deflection of the whole span. The bending strength at rupture (MOR) was estimated following Equation (8).

(6) $${\mathrm{MOE}}_{S.\mathrm{local}}=\frac{a\cdot l_{1}^{2}\cdot \left(F_{2}-F_{1}\right)}{16\cdot I\cdot \left(w_{2}-w_{1}\right)}$$

(7) $${\mathrm{MOE}}_{S.\mathrm{global}}=\frac{l^{3}\cdot \left(F_{2}-F_{1}\right)}{b\cdot h^{3}\cdot \left(y_{2}-y_{1}\right)}\cdot \left(\frac{3\cdot a}{4\cdot l}-{\left(\frac{a}{l}\right)}^{3}\right)$$

(8) $$\mathrm{MOR}=\frac{a\cdot \left(\frac{F_{\max}}{2}\right)}{W}$$

where *a* is distance between loading and the nearest bearing point (*a* = 6·*h*), *l* is total distance between the bearing points (*l* = 18·*h*), *l*_1_ is length of central gauge (*l*_1_ = 5·*h*), *b* is board thickness and *h* is the board width. Furthermore, *F*_2_ − *F*_1_ represents an increment of applied load derived from the first linear part of the load-deformation curve (*F*_1_ = 0.1·*F*_max_ and *F*_2_ = 0.4·*F*_max_), *F*_max_ is the maximum load, and *w*_2_ − *w*_1_ and *y*_2_ − *y*_1_ are deformation increments corresponding to *F*_2_ − *F*_1_ (Figure 3). The second area moment of each board (*I* in Equation (6)) was in turn calculated as

(9) $$I=\frac{b\cdot h^{3}}{12}$$

and *W* is the section modulus obtained as

(10) $$W=\frac{b\cdot h^{2}}{6}.$$

Equation (7) is a simplification of the corresponding equation for MOE_S.global_ stated in EN408, as the shear modulus was set to infinity according to the EN 384 strength class allocation procedure and is therefore omitted from the equation. MOE_S.local_ and MOE_S.global_ were adjusted to an MC of 12% following the EN 384 standard [29]:

(11) $${\mathrm{MOE}}_{S.\mathrm{adjust}}={\;\mathrm{MOE}}_{S}+{\mathrm{MOE}}_{S}\cdot 0.01\cdot \left({\mathrm{MC}}_{\mathrm{sample}}-12\right)\;\mathrm{for}\;\mathrm{samples}\;\mathrm{with}\;\mathrm{MC}\;<\;18\%$$

(12) $${\mathrm{MOE}}_{S.\mathrm{adjust}}={\;\mathrm{MOE}}_{S}+{\mathrm{MOE}}_{S}\cdot 0.01\cdot \left(18-12\right)\;\mathrm{for}\;\mathrm{samples}\;\mathrm{with}\;\mathrm{MC}\;>\;18\%.$$

MOR was adjusted with respect to board dimension as

(13) $${\mathrm{MOR}}_{\mathrm{adjust}}=\frac{\mathrm{MOR}}{k_{h}}$$

where the correction factor *k_h_* equals 1 for *h* > 150, whilst for *h* < 150 it is calculated as

(14) $$k_{h}=\;min\{\begin{array}{c} {\left(\frac{150}{h}\right)}^{0.2}\\ 1.3 \end{array}.$$

### 2.5. Statistical Analysis

Using statistical package ASReml 4 [30], the response variables (Table 1) were fitted into the linear mixed model:

(15) $$y_{ijj’k}=\mu +B_{i}+P_{j}+P_{j’}+F_{jj’}+e_{ijj’k}$$

where *y_ijj’k_* is a value for *k*th offspring of *j*th and *j’*th parents growing in *i*th block, *B* is fixed effect of block, *P*, *F* and *e* are random effects of parent, family and residual, respectively, and *μ* is the overall mean of a given variable. The model above was used in a bivariate setting where the trait of interest was consistently paired with the resistograph-based density. DEN_RES_ was always included because it was assessed on all surviving trees in the progeny test (1896), thus potentially improving parameter estimates of the other traits by better accounting for mortality, missing values and potential selective biases due to the necessary selection of sawmillable trees [31]. Also since wane wider than 2 cm was present on ca 30% of the boards, maximum wane depth was added to the model as a fixed covariate for all variables measured on boards. Averaged A- and B-board wane depths were used for the traits measured on pairs of boards while B-board wane depth was used for the traits measured just on the respective set of boards.

Assuming that epistatic genetic variation was absent, the individual-tree narrow-sense heritability ($h_{i}^{2}$), broad-sense heritability ($H_{i}^{2}$) and dominance ratio ($d_{i}^{2}$) for each trait were estimated as

(16) $$h_{i}^{2}=\frac{\sigma_{A}^{2}}{\sigma_{P}^{2}}$$

(17) $$H_{i}^{2}=\frac{\sigma_{G}^{2}}{\sigma_{P}^{2}}$$

(18) $$d_{i}^{2}=\frac{\sigma_{D}^{2}}{\sigma_{P}^{2}}$$

where $\sigma_{A}^{2}$, $\sigma_{P}^{2}$, $\sigma_{G}^{2}$, and $\sigma_{D}^{2}$ are additive genetic, phenotypic, genotypic and dominance variance components, respectively, obtained from the bivariate analyses based on Equation (15). Genetic and phenotypic variances were in turn estimated as follows:

(19) $$\sigma_{A}^{2}=4\sigma_{p}^{2}$$

(20) $$\sigma_{G}^{2}=4(\sigma_{p}^{2}+\sigma_{f}^{2})$$

(21) $$\sigma_{D}^{2}=4\sigma_{f}^{2}$$

(22) $$\sigma_{P}^{2}=2\sigma_{p}^{2}+\sigma_{f}^{2}+\sigma_{e}^{2}$$

where $\sigma_{p}^{2}$, $\sigma_{f}^{2}$ and $\sigma_{e}^{2}$ are model variance components for parental, family and residual model terms, respectively. Standard errors were obtained using Taylor series expansion [30]. For comparing variances of different traits, coefficients of variation were estimated as

(23) $$CV_{i}=\frac{\sigma_{i}}{\bar{x}}\cdot 100$$

where $\sigma_{i}$ represents phenotypic ($\sigma_{P}$), additive ($\sigma_{A}$) and genotypic ($\sigma_{G}$) standard deviations for respective phenotypic ($CV_{P}$), additive genetic ($CV_{A}$) and genotypic ($CV_{G}$) coefficients of variation, and $\bar{x}$ is a trait’s mean. 

Finally, bivariate and trivariate analyses of the model in Equation (15) were carried out in order to estimate phenotypic and genetic correlation coefficients (*r_xy_*) between pairs of traits (*x* and *y*) as

(24) $$r_{xy}=\frac{\sigma_{xy}}{\sqrt{\sigma_{x}^{2}\times \sigma_{y}^{2}}}$$

where $\sigma_{x}^{2}$ and $\sigma_{y}^{2}$ are phenotypic or additive genetic variances for traits *x* and *y*, respectively, and $\sigma_{xy}$ is phenotypic or additive genetic covariance between traits *x* and *y*. In these bivariate and trivariate analyses, the two traits of interests were consistently accompanied by DEN_RES_ according to the same methodology already described above for variance and heritability estimation. 

Genetic gain ($G_{A_{x}}$) for direct selection was estimated [32] as

(25) $$G_{A_{x}}=ih_{x}^{2}\sigma_{P_{x}}=ih_{x}\sigma_{A_{x}}$$

and correlated response ($CR_{A_{y}}$) of a target trait *y* as a result of the selection for measurement trait *x* was calculated as

(26) $$CR_{A_{y}}=ih_{x}h_{y}r_{A_{xy}}\sigma_{P_{y}}=ih_{x}r_{A_{xy}}\sigma_{A_{y}}$$

where *i* is selection intensity, $h_{x}^{2}$ is narrow-sense heritability for trait *x*,$\;h_{x}$ and $h_{y}$ are squared roots of narrow-sense heritabilities for selection trait *x* and target trait *y*, respectively, $r_{A_{xy}}$ is additive genetic correlation between traits *x* and *y* and $\sigma_{P_{x}}$, $\sigma_{P_{y}}$, $\sigma_{A_{x}}$,$\;\sigma_{A_{y}}$ are phenotypic and additive genetic standard deviations for traits *x* and *y*.

## 3. Results

### 3.1. Range and Mean of Phenotypic Measurements

Variables and their descriptive statistics are summarized in Table 1 and Table 2, respectively. Since the destructively measured MOE_S.local_ and MOE_S.global_ are the target traits for breeders, they were used as benchmark variables for evaluation of different non-destructive stiffness assessments. MOE_S.local_ and MOE_S.global_ ranged from 4.17 to 15.43 GPa and from 4.18 to 13.03 GPa, respectively, with mean values of 8.50 GPa and 7.90 GPa. Compared with the static target MOE_S_, the ranges and means were slightly lower for MOE_BOARD_, slightly higher for MOE_TREE_ and substantially higher for MOE_LOG_ with a maximum value of 38.16 GPa. Acoustic velocity measured on standing trees (VEL_HIT_) was a little higher than acoustic velocity measured on boards (VEL_MTG_), whilst that measured on logs (VEL_FAK_) was almost twice as high. Wood density of boards calculated as mass over volume (DEN_VOL_) ranged from 383 to 555 kg·m^−3^, with the mean value being 462 kg·m^−3^, whilst the adjusted wood density measured non-destructively on standing trees by the Resistograph (DEN_RES_) exhibited a wider range as well as a higher mean value (+12.9%). As expected, mean DBH was substantially (17.9%) higher for trees selected for the sawmill study compared to the unselected ones (Table A1). Also, the means of other traits measured on standing trees were higher but only to a slight degree (0.8–2.6%). Except for STR, all pairs of means were significantly different (*p* < 0.05).

### 3.2. Variation and Heritability

Coefficients of phenotypic, additive genetic and genotypic variation as well as individual-tree narrow- and broad-sense heritabilities are shown in Table 2. Among the variables included in the study, MOR exhibited the highest phenotypic variation (24.0%) and was followed by different MOE estimates and DBH. On the other hand, all VEL estimates and DEN_VOL_ had the lowest phenotypic variation estimates (5.5–7.2%). Estimates of additive genetic variation were overall limited, ranging from 1.5% for VEL_HIT_ to 11.7% for DBH, whilst genotypic variation estimates were higher, ranged from 3.0% for VEL_FAK_ to 16.9% for DBH.

Individual-tree narrow-sense heritabilities for the three structural target traits MOE_S.local_, MOE_S.global_ and MOR were rather low (0.08–0.14), and only a little higher values (0.17–0.26) were obtained for non-destructively estimated MOE. Heritability of VEL_HIT_ (0.05) was very low compared to those of VEL_FAK_ and VEL_MTG_ (0.20 and 0.24, respectively). The highest estimates were obtained for DEN_VOL_ and DEN_RES_ (0.34 and 0.40, respectively); those for DBH and STR were moderate (0.24 and 0.28, respectively). Broad-sense heritabilities ranged from 0.28 for VEL_HIT_ to 0.62 for DEN_RES_ and were substantially and consistently higher than the corresponding $h_{i}^{2}$-estimates for all traits. Consequently, the estimated dominance ratios ($d_{i}^{2}$), expressing the proportion of phenotypic variance due to dominance effects ($\sigma_{D}^{2}/\sigma_{P}^{2}$), were also considerable in comparison to the $h_{i}^{2}$-estimates and ranged between 0.09 and 0.24 for VEL and between 0.14 and 0.33 for MOE.

The proportion of additive genetic to genotypic variance ($\sigma_{A}^{2}/\sigma_{G}^{2}$) averaged 0.49 (Table 2). The lowest $\sigma_{A}^{2}/\sigma_{G}^{2}$ ratios were obtained for VEL_HIT_ measured on standing trees and the target MOE_S.local_ and MOE_S.global_ (0.18, 0.30 and 0.20, respectively), while rather high values were obtained for VEL_FAK_ measured on felled logs and DEN_VOL_ (0.70 and 0.71, respectively).

### 3.3. Phenotypic (r_P_) and Additive Genetic (r_A_) Correlations

Phenotypic and additive genetic correlations of destructively measured MOE_S.local_, MOE_S.global_ and MOR with different non-destructively assessed estimates of MOE, VEL, DEN and with growth traits are presented in Table 3. Correlations among destructively obtained traits and among all other traits are presented in Table 4 and Table 5, respectively. Additive genetic correlations of destructively measured target MOE_S.local_ and MOE_S.global_ with different indirect estimates of MOE were strong (0.70–0.98), whereas phenotypic correlations between the same traits ranged from moderate to strong (0.46–0.83). Correlations of MOE_S.local_ and MOE_S.global_ with VEL showed a similar pattern (*r*_A_ = 0.65–0.97 and *r*_P_ = 0.32–0.75). The strongest genetic correlations with both measures of static MOE were obtained for wood stiffness assessed on sawn boards using acoustic resonance combined with volumetric wood density (MOE_BOARD_; 0.98 and 0.95) and acoustic velocity measured on standing trees (VEL_HIT_; 0.96 and 0.97). VEL_HIT_ also exhibited the strongest genetic correlation with MOR (0.99) and was closely followed by stiffness assessed on logs and standing trees (MOE_LOG_ and MOE_TREE_; 0.94 and 0.90 respectively) and by the resistograph density alone (DEN_RES_; 0.86). Among the DEN estimates, DEN_RES_ exhibited the strongest genetic correlations with all the structural target traits (0.60–0.86), whilst the corresponding phenotypic correlations were moderate (0.43–0.48). Genetic and phenotypic correlations of destructively measured traits with STR were positive, with moderate (0.55–0.66) and weak (0.12–0.17) magnitudes, respectively. On the other hand, negative genetic and phenotypic correlations were obtained between DBH and all static MOE and MOR traits with estimates ranging from −0.03 to −0.57. MOE_S.local_ and MOE_S.global_ were strongly correlated at both the genetic and phenotypic levels (*r*_A_ = 0.96 and *r*_P_ = 0.93, Table 4). Their correlations with MOR were slightly lower.

All correlations among non-destructively measured indirect MOE traits were moderate to strong (0.55–0.97, Table 5); nevertheless, the relationship between MOE_TREE_ and MOE_LOG_ should be interpreted with caution as both estimates were calculated using the same DEN_RES_ thereby making them susceptible to autocorrelation. For the same reason, the strong correlations between VEL measures and their respective MOE estimates (0.71–0.92) might be somewhat inflated. Genetic correlations among different VEL estimates were strong (0.74–0.94) whilst the phenotypic were only moderate (0.36–0.60). The strongest genetic correlation was found between acoustic velocities measured on trees (VEL_HIT_) and logs (VEL_FAK_) (0.94). Genetic correlations among VEL measures and unrelated indirect MOE estimates varied from moderate (0.32 between VEL_MTG_ and MOE_TREE_) to strong (0.95 between VEL_HIT_ and MOE_LOG_). Correlations between DEN_VOL_ and DEN_RES_ were strong (*r*_A_ = 0.75 and *r*_P_ = 0.72). Genetic correlations between STR and non-destructively measured wood traits were effectively close to zero (0.08–0.33), except for those obtained for traits measured on boards (VEL_MTG_ and MOE_BOARD_), which were moderately positive (0.59 and 0.61) like the corresponding STR genetic correlations with structural target traits. Genetic correlations of DBH with the wood traits varied substantially from weakly positive (DEN_RES_; *r*_A_ = 0.24) to moderately negative (MOE_BOARD_; *r*_A_ = −0.49) and were at the same time associated with rather high standard errors.

### 3.4. Correlated Response to Selection

Correlated response of economically important but hard−to−measure traits (target traits) to selection based on easy-to-measure traits (selection traits) is shown in Table 6. Selection for DBH resulted in genetic gain in the trait itself (15.2%) but in genetic losses for all structural wood target traits (from −0.4 to −3.4%). On the other hand, selection for STR, DEN_RES_ or MOE_TREE_ led to fair improvements of structural wood traits without any genetic loss in DBH. The highest genetic gains for most of the target traits were achieved by selection for DEN_RES_, resulting in ca 5%, 5%, 8%, 7% and 13% increases in DBH, DEN_VOL_, MOE_S.local_, MOE_S.global_ and MOR, respectively.

## 4. Discussion

Stiffness and strength are important wood quality properties that predetermine the suitability of sawn wood for construction purposes. The possibility of their non-destructive assessment offers an opportunity to select trees for wood quality improvement, to optimize silvicultural practices towards higher wood quality, or to effectively assort wood sources according to different end-use requirements.

Destructively assessed local (MOE_S.local_) and global (MOE_S.global_) moduli of elasticity and modulus of rupture (MOR) were set as benchmarks for evaluation of the ability of non-destructive methods to accurately assess stiffness and strength of the final product, represented by sawn boards, at different stages of wood processing, namely, on standing trees, logs and the boards. In order to realize genetic gains through selection and breeding, the most important task is to predict properties of a final product from measurements on young standing trees.

### 4.1. Phenotypic and Genetic Variation in Wood Stiffness and Acoustic Velocity

Phenotypic values for acoustic velocity (VEL_FAK_) measured on logs and, consequently, also values for modulus of elasticity (MOE_LOG_) calculated from VEL_FAK_ were substantially higher compared to other VEL and MOE traits measured either on sawn boards or on standing trees (Table 2). A number of studies, however, reported a lower acoustic velocity measured on logs compared to that measured on standing trees [17,33]. One possible explanation for the discrepancy in VEL values could be that the second resonance frequency, instead of the first one, was recorded on logs. In such a case, resonance frequency should be divided by two, which would result in a half VEL_FAK_ value. Nevertheless, from the quantitative genetics point of view, it can be considered just a matter of different scaling, with no influence on further analyses. This notion is supported by the strong genetic correlations of MOE_LOG_ with the benchmark target traits MOE_S.local_, MOE_S.global_ and MOR (0.84–0.94).

*CV_P_* coefficients for different non-destructive MOE estimates measured on standing Scots pine trees, comparable to those obtained in this study, were reported by [34]. Similar *CV_P_* but higher *CV_A_* were estimated for MOE in Norway spruce (*CV_P_* ≈ 17% and *CV_A_* ≈ 10%) [35]. Both *CV_P_* and *CV_A_* coefficients for VEL estimates were rather low in this study; *CV_P_* of the same magnitude was reported e.g., by [35,36,37], whereas a higher *CV_A_* was reported by [35,38,39].

### 4.2. Narrow-Sense Heritability

Individual-tree narrow-sense heritability estimates for VEL (0.05–0.24) and MOE (0.08–0.26), reported in Table 2, were weak but in most cases still appreciable (>0.10). Low heritabilities for benchmark local and global static MOE (0.11 and 0.08, respectively) and MOR (0.14) were a little lower than those reported for Norway spruce sawn boards (0.23 for MOE_S.local_ and 0.21 for MOR) [40]. On the other hand, heritabilities for static MOE and MOR calculated based on destructive testing of small clear specimens were found to be moderate in a number of conifer species, e.g., 0.53 and 0.54 for radiata pine [7] or 0.44 and 0.60 for hybrid larch [9], respectively. It appears that direct measurements of MOE_S_ and MOR on small clear-wood samples result in higher heritabilities compared to measurements carried out on sawn boards.

Narrow-sense heritability for acoustic velocity measured on standing trees (VEL_HIT_) was very low (0.05) compared to other studies. Generally, moderate heritabilities (~0.38) were reported for conifer tree species [7,8,34,41,42,43]. Nevertheless, a low heritability was estimated e.g., for Norway spruce (0.15) [35] or Douglas-fir (0.14) [37]. As a likely consequence of the low heritability for VEL_HIT_ in this study, the standing-tree modulus of elasticity (MOE_TREE_) calculated from VEL_HIT_ also showed a rather low heritability (0.22). Similar results were reported e.g., for lodgepole pine (*Pinus contorta* Douglas ex Loudon) (0.20) [44], but higher estimates have been reported too, e.g., for Norway spruce (0.31) or Scots pine (0.45) [34,35], respectively.

Compared with VEL_HIT_, higher heritabilities (0.20 and 0.24) were obtained for acoustic velocity measured on felled logs (VEL_FAK_) and sawn boards (VEL_MTG_), respectively (Table 2). Nevertheless, heritabilities of acoustic velocity measured on logs of other coniferous species were double (≈0.46) [7,8,38]. Heritabilities of MOE_LOG_ (0.26) and MOE_BOARD_ (0.17) were comparable to heritability of MOE_TREE_ and higher than those of benchmark MOE_S.local_ and MOE_S.global_. Closer MOE_BOARD_ heritability (0.23) was obtained for Norway spruce by [12].

Narrow-sense heritabilities for wood density assessed by the volumetric approach (DEN_VOL_) and Resistograph (DEN_RES_) were both higher (0.34 and 0.40, respectively) than the $h_{i}^{2}$-estimates for any other trait assessed in this study. A similar heritability of DEN_VOL_ was also observed, e.g., in Norway spruce (0.44) [12] whilst a stronger heritability was reported for radiata pine (0.70) [7]. A moderate heritability of DEN_RES_ was found in another study of Scots pine (0.43) [24], whereas it was a little weaker in loblolly pine (*Pinus taeda* L.) (0.28) [42].

Heritability of stem straightness (STR) varied from low to high [7,45,46,47] in other studies with pine species. The results may however have been influenced, aside from other factors, by a different number of classes used for visual scoring [48].

### 4.3. Additive and Non-Additive Variance

In the current study, all VEL, MOE and MOR estimates showed a low level of additive genetic control. Whilst narrow-sense heritabilities ($h_{i}^{2}$) for these traits were low, their broad-sense counterparts ($H_{i}^{2}$) were more than the double magnitudes for seven out of nine traits thus indicating the presence of non-additive genetic variance. Substantial non-additive effects have previously been reported for growth traits, e.g., in Norway spruce [49], black spruce (*Picea mariana* [Mill.] B.S.P.) [50], radiata pine [51] or *Eucalyptus globulus* [52], unlike wood quality traits, among which low or no non-additive effects were observed. No dominance effects were also reported for MOE and MOR in hybrid larch [9] and for squared acoustic velocity in juvenile wood of Douglas-fir and western hemlock (*Tsuga heterophylla* (Raf.) Sarg.) [53]. On the other hand, an average $\sigma_{A}^{2}/\sigma_{G}^{2}$ ratio of 0.75 was estimated for MOE_LOG_, modelled from standing-tree VEL, in radiata pine [54]. In summary, the considerable non-additive variances for wood traits estimated in this study diverges substantially from most such estimates previously published for tree species [55]. If interpreted at face value, our results suggest that vegetative propagation would probably be a more efficient approach in deployment for improved structural wood quality as such methods would be better capable of capturing both additive and non-additive genetic variance.

Nevertheless, pedigree errors can falsely inflate family variance ($\sigma_{f}^{2}$) and thereby confound additive and non-additive effects [52]. Both the non-additive effects and pedigree errors can result in lower $h_{i}^{2}$ and $\sigma_{A}^{2}/\sigma_{G}^{2}$, and higher $d_{i}^{2}$. Since we did not have the possibility to verify the pedigree using genetic markers and relied on pedigree records as indicated in the breeding program, we cannot exclude with certainty the possibility that our data contain pedigree errors.

### 4.4. Predictability of Sawn-Board Quality at Different Stages along the Wood Processing Chain

In this study, strong additive genetic (0.70–0.90) and moderate phenotypic (0.40–0.52) correlations of MOE_TREE_ with the three benchmark traits were revealed. A lower genetic correlation between destructively measured MOE_S_ and MOE_TREE_ was reported for Douglas-fir (*r*_A_ = 0.57) [8]. Phenotypic correlations between MOE_S_ and MOE_TREE_ of a similar magnitude (0.45) were obtained for dimensional lumber of Douglas-fir [8,56], whereas stronger correlations were found for small clear-wood samples of western hemlock and Sitka spruce (*Picea sitchensis* (Bong.) Carr.) (*r*_P_ = 0.66) [57], radiata pine (*r*_P_ = 0.62) [58] and loblolly pine (*r*_P_ = 0.81) [21]. It appears that measurements taken from small clear-wood samples generate stronger genetic and phenotypic correlations [59]. Small samples are free from strength-reducing features such as knots or cracks, and provide a good image of mechanical properties of the wood itself. On the other hand, full-size boards, used e.g., for construction purposes, offer a more complex and realistic view of the wood material including all its “imperfections”. Moreover, correlation estimates may differ depending on the way of calculating the dynamic MOE_TREE_. Most commonly, squared TOF-based acoustic velocity measured on standing trees is multiplied by green density, which can be represented by volumetric green density [8,21,57], constant green density of 1000 kg·m^−3^ [58] or by resistograph green density (DEN_RES_), as in this study. It is quite easy to estimate wood density volumetrically; nevertheless, it is not feasible in operational scale. Given the results of our study and of others, it therefore appears that resistograph as well as constant density can be used as suitable proxies for the volumetric density, resulting in accurate MOE_TREE_ estimates [34].

In this study, acoustic velocity measured on standing trees (VEL_HIT_) exhibited very strong genetic (0.96–0.99) but rather weak phenotypic correlations (0.23–0.37) with structural target traits. Lower genetic (0.69) but closer phenotypic correlations (0.47) were observed in radiata pine [7] or Douglas-fir (*r*_A_ = 0.53, *r*_P_ = 0.35) [8]. On the other hand, a considerably higher phenotypic correlation (0.72) was reported for a different study on Scots pine growing in Scotland [17]. With respect to correlations between standing-tree VEL and MOR, [7] reported genetic correlations (0.68) for radiata pine weaker than those of this study whilst phenotypic correlations were close (0.40) to ours. A phenotypic correlation (0.77) stronger than ours was again reported for Scots pine in Scotland [17].

Strong additive genetic (0.84–0.94) and moderate phenotypic (0.50–0.57) correlations between stiffness assessed on logs (MOE_LOG_) and the benchmark MOE_S.local_, MOE_S.global_ and MOR (Table 3) were in good accordance with studies on hybrid larch [9], Douglas-fir [9] and Jack pine (*Pinus banksiana* Lamb.) [60]. Likewise, genetic and phenotypic correlations between acoustic velocity measured on felled logs (VEL_FAK_) and the benchmark traits were strong (0.72–0.92) and moderate (0.43–0.55), respectively. Comparable estimates were reported also for radiata pine [7], Douglas-fir [8] or *Eucalyptus nitens* [10].

Genetic correlations of MOE_S_ with wood density estimates varied from somewhat stronger (0.60, 0.74) in the case of DEN_RES_ to weaker (0.34, 0.48) in the case of DEN_VOL_ (Table 3). In other studies, they varied from weak (0.25) [61] and moderate (≈0.55) [10,41] to strong (>0.70) [7,9]. The strong genetic correlation of MOR with DEN_RES_ (0.86) was in congruence with other studies that estimated the relationship between MOR and wood density [7,9,41,61]. However, in our study, the genetic correlation between MOR and DEN_VOL_ was only moderate (0.66). Besides, strong correlations (0.75–0.80) between destructively assessed stiffness (MOE_S_) and strength (MOR) (Table 4) were consistent with other studies [41,61].

Finally, considerable genetic correlations of stem straightness with stiffness-related traits measured on full sized sawn boards (~0.6) confirm that the orientation of wood fibers has a great effect on stiffness and strength [27]. In contrast, weak negative correlations with MOE_S_ and MOR measured destructively on small clear-wood samples (−0.22 ± 0.42 and −0.19 ± 0.41, respectively) were observed in radiata pine [7].

Taken together, the results suggest that all three acoustic-based MOE measures included in this study (MOE_TREE_, MOE_LOG_ and MOE_BOARD_) as well as all acoustic velocities (VEL_HIT_, VEL_FAK_ and VEL_MTG_) are good proxies for sawn-board stiffness (MOE_S_). Moreover, MOE_TREE_, MOE_LOG_, VEL_HIT_, VEL_FAK_ and DEN_RES_ provide good prediction of sawn-board strength (MOR).

### 4.5. Relationship between Growth and Structural Wood Traits

Phenotypic and additive genetic correlations of DBH with the benchmark structural traits (MOE_S.local_, MOE_S.global_ and MOR, Table 3) and other wood traits measured on sawn boards (MOE_BOARD_, VEL_MTG_ and DEN_VOL_, Table 5) were weakly to moderately negative (−0.03 to −0.65). These results are in congruence with those reported in a number of other studies: most of the genetic correlations between DBH and MOEs were negative, either weak [9,41,56], moderate [7,61] or varying by DBH measurement age [40,55]. In exception, weak positive correlations between DBH and MOEs were observed by [8]. Additive genetic correlations between DBH and MOR ranged from none [61] through weakly negative [41] to moderately negative [7].

On the other hand, wood quality traits measured on standing trees (MOE_TREE_, VEL_HIT_ and DEN_RES_) exhibited weak positive correlations with DBH, both at the genetic (0.14–0.24) and phenotypic (0.09–0.10) levels. Additive genetic correlations between DBH and standing-tree VEL varied in published studies from weakly positive [62] and none [43] through weakly negative [34,35,56] up to strongly negative [7]. It should be noted that, in this study, measurements on standing trees were taken for all living trees in the field trial (1896) but only about a quarter of those (with DBH > 15 cm) were harvested and processed into boards. Aside from the lower diameter, the unselected trees (1400) also exhibited a somewhat lower density and stiffness compared to the selected trees (Table A1). This would correspond with the fact that trees with a lower diameter have a higher proportion of juvenile wood [63] and with the positive correlations estimated in this study between DBH and wood traits assessed on standing trees. Nevertheless, due to the relatively high standard errors associated with additive genetic correlation estimates, which were also reported in other studies [7,8,9,40,41], the results should be interpreted with caution.

### 4.6. Implications for Breeding

Despite the low narrow-sense heritabilities, fair improvements of structural target traits were attained in this study by applying indirect selection. The essential factor for such an achievement is a fair variation of the target traits combined with a high heritability of the selection trait and/or high genetic correlation between the target and selection traits. Of the possible selection traits, i.e., those that are measurable non-destructively on standing trees, MOE_TREE_, DEN_RES_ and STR were indicated a suitable for structural wood quality improvement. MOE_TREE_ exhibited strong genetic correlations with the target traits, although its heritability was rather low. On the other hand, DEN_RES_ showed a high heritability as well as decently strong genetic correlations. Surprisingly, STR also turned out well as a selection trait owing to its moderate heritability and correlations. On the contrary, despite very strong genetic correlations with the target traits, VEL_HIT_ did not perform as anticipated because its heritability was extremely low.

The results suggest that DEN_RES_ would be the best choice for indirect improvement of board stiffness, strength and density. However, the potential of STR should also be considered. STR is the main determinant of a log’s value as it affects most of the processing steps as well as the proportion of sawmill recovery. Moreover, this study revealed reasonably strong relationships between STR and board stiffness and strength, which may speed up phenotypic selection procedures because scoring of STR is fast and does not require any costly tools.

## 5. Conclusions

This study aimed to evaluate the ability of non-destructive acoustic-based tools, applied on standing trees, felled logs and sawn boards of Scots pine, to accurately assess their destructively measured static wood stiffness and strength from a tree breeding point of view. The results suggest that all three acoustic-based stiffness (MOE_TREE_, MOE_LOG_ and MOE_BOARD_) as well as acoustic velocity measures (VEL_HIT_, VEL_FAK_ and VEL_MTG_) provide a good estimate of sawn-board stiffness (MOE_S_). Moreover, MOE_TREE_, MOE_LOG_, VEL_HIT_, VEL_FAK_ and resistograph wood density measured on standing trees (DEN_RES_) well reflected sawn-board strength (MOR). In the studied material, all VEL, MOE and MOR traits exhibited low levels of additive genetic control ($h_{i}^{2}$ ranged from 0.05 to 0.26). However, because of the reasonably high heritabilities for selective traits MOE_TREE_, DEN_RES_ or STR coupled with their relatively strong genetic correlations with target structural wood traits, selective breeding based on any of them (or combination of either MOE_TREE_ and STR or DEN_RES_ and STR) would result in a desirable increase in sawn-board quality (stiffness, strength and density).

## Figures and Tables

**Table 1:** List of trait variables assessed for the material at the field age of 38 years.

Trait	Units	Description
DBH	cm	Diameter at breast height
STR	-	Stem straightness (1–9 with 9 as the most straight tree)
DEN_RES_	kg·m^−3^	Adjusted mean density number measured on standing trees by Resistograph
DEN_VOL_	kg·m^−3^	Volumetric density of boards
VEL_HIT_	km·s^−1^	Acoustic velocity measured on standing trees by Hitman
VEL_FAK_	km·s^−1^	Acoustic velocity calculated from resonance frequency measured on logs by Fakopp (Equation (3))
VEL_MTG_	km·s^−1^	Acoustic velocity calculated from resonance frequency measured on boards by MTG grader (Equation (5))
MOE_TREE_	GPa	Dynamic modulus of elasticity calculated from VEL_HIT_ and DEN_RES_ (Equation (1))
MOE_LOG_	GPa	Dynamic modulus of elasticity calculated from VEL_FAK_ and DEN_RES_ (Equation (2))
MOE_BOARD_	GPa	Dynamic modulus of elasticity calculated from VEL_MTG_ and DEN_VOL_ (Equation (4))
MOE_S.local_	GPa	Local static modulus of elasticity (Equation (6))
MOE_S.global_	GPa	Global static modulus of elasticity (Equation (7))
MOR	MPa	Modulus of rupture (Equation (8))

**Table 2:** Descriptive statistics of the studied traits—number of observations (*n*), minimum, maximum, mean, standard deviation (*SD*), coefficient of phenotypic (*CV_P_*), additive (*CV*_A_) and genotypic (*CV_G_*) variation, individual-tree narrow-sense ($h_{i}^{2}$), and broad-sense ($H_{i}^{2}$) heritability, dominance ratio ($d_{i}^{2}$), and ratio of additive and genotypic variance ($\sigma_{A}^{2}/\sigma_{G}^{2}$) (standard errors are in parentheses).

*Trait*	*Units*	*n*	*Min*	*Max*	*Mean*	*SD*	*CV_P_*	*CV_A_*	*CV_G_*	$h_{i}^{2}$	$H_{i}^{2}$	$d_{i}^{2}$	$\sigma_{A}^{2}/\sigma_{G}^{2}$
DBH	cm	1896	6.10	30.40	17.22	4.10	23.75	11.69	16.92	0.24 (0.08)	0.51 (0.09)	0.27 (0.07)	0.48
STR	-	1896	2.00	9.00	7.84	0.79	9.92	5.22	6.35	0.28 (0.08)	0.41 (0.09)	0.13 (0.05)	0.68
DEN_RES_	kg·m^−3^	1892	337.10	694.50	521.47	53.09	10.29	6.50	8.12	0.40 (0.11)	0.62 (0.11)	0.22 (0.07)	0.64
DEN_VOL_	kg·m^−3^	494	382.70	555.15	462.08	28.16	6.13	3.60	4.26	0.34 (0.11)	0.48 (0.12)	0.14 (0.09)	0.71
VEL_HIT_	km·s^−1^	1874	3.11	4.99	4.22	0.28	6.67	1.49	3.55	0.05 (0.03)	0.28 (0.07)	0.23 (0.07)	0.18
VEL_FAK_	km·s^−1^	486	6.00	8.10	6.75	0.37	5.51	2.50	2.98	0.20 (0.09)	0.29 (0.13)	0.09 (0.12)	0.70
VEL_MTG_	km·s^−1^	496	2.86	4.67	3.88	0.28	7.18	3.48	4.97	0.24 (0.10)	0.48 (0.15)	0.24 (0.15)	0.49
MOE_TREE_	GPa	1873	3.56	15.99	9.36	1.79	19.24	8.99	13.61	0.22 (0.08)	0.50 (0.09)	0.28 (0.08)	0.44
MOE_LOG_	GPa	494	10.42	38.16	24.27	4.30	17.91	9.14	11.37	0.26 (0.09)	0.40 (0.12)	0.14 (0.10)	0.65
MOE_BOARD_	GPa	495	2.77	10.77	6.90	1.33	19.03	7.84	11.76	0.17 (0.08)	0.38 (0.13)	0.21 (0.13)	0.44
MOE_S.local_	GPa	494	4.17	15.43	8.50	2.03	23.16	7.67	13.89	0.11 (0.07)	0.36 (0.13)	0.25 (0.15)	0.30
MOE_S.global_	GPa	495	4.18	13.03	7.90	1.54	19.12	5.43	12.20	0.08 (0.07)	0.41 (0.14)	0.33 (0.15)	0.20
MOR	MPa	495	16.25	56.37	31.81	6.93	23.96	8.96	13.10	0.14 (0.07)	0.30 (0.13)	0.16 (0.13)	0.47

**Table 3:** Additive genetic (*r*_A_) and phenotypic (*r*_P_) correlations of destructively measured target traits (MOE_S.local_, MOE_S.global_ and MOR) with different dynamic moduli of elasticity, acoustic velocities, densities and growth traits (standard errors in parentheses).

	Genetic Correlations			Phenotypic Correlations		
	MOE_S.local_	MOE_S.global_	MOR	MOE_S.local_	MOE_S.global_	MOR
**MOE_TREE_**	**0.70** (0.22)	**0.78** (0.20)	**0.90** (0.15)	0.46 (0.04)	0.52 (0.03)	0.40 (0.04)
**MOE_LOG_**	**0.84** (0.18)	**0.85** (0.21)	**0.94** (0.13)	0.53 (0.03)	0.57 (0.03)	0.50 (0.03)
**MOE_BOARD_**	**0.98** (0.07)	**0.95** (0.08)	**0.62** (0.23)	0.77 (0.02)	0.83 (0.01)	0.63 (0.03)
**VEL_HIT_**	**0.96** (0.24)	**0.97** (0.23)	**0.99** (0.23)	0.32 (0.04)	0.37 (0.04)	0.23 (0.04)
**VEL_FAK_**	**0.92** (0.16)	**0.75** (0.21)	**0.72** (0.22)	0.50 (0.04)	0.55 (0.03)	0.43 (0.04)
**VEL_MTG_**	**0.78** (0.16)	**0.65** (0.23)	0.26 (0.33)	0.70 (0.02)	0.75 (0.02)	0.54 (0.03)
**DEN_RES_**	**0.60** (0.27)	**0.74** (0.27)	**0.86** (0.16)	0.43 (0.04)	0.48 (0.03)	0.44 (0.04)
**DEN_VOL_**	0.34 (0.31)	0.48 (0.32)	**0.66** (0.22)	0.42 (0.04)	0.47 (0.04)	0.42 (0.04)
**DBH**	−0.34 (0.30)	−0.47 (0.28)	−0.03 (0.32)	−0.56 (0.03)	−0.57 (0.03)	−0.38 (0.04)
**STR**	**0.66** (0.22)	**0.57** (0.28)	**0.55** (0.23)	0.16 (0.04)	0.17 (0.04)	0.12 (0.04)

Note: Genetic correlations with magnitudes greater than two times their estimation error are highlighted in bold. All phenotypic correlations were significant at the 5% level.

**Table 4:** Additive genetic (*r*_A_, above diagonal) and phenotypic (*r*_P_, below diagonal) correlations among destructively measured variables (standard errors in parentheses).

	MOE_S.local_	MOE_S.global_	MOR
MOE_S.local_		**0.96** (0.04)	**0.80** (0.16)
MOE_S.global_	0.93 (0.01)		**0.78** (0.19)
MOR	0.75 (0.02)	0.78 (0.02)	

Note: Genetic correlations with magnitudes greater than two times their estimation error are highlighted in bold. All phenotypic correlations were significant at the 5% level.

**Table 5:** Additive genetic (*r*_A_, above diagonal) and phenotypic (*r*_P_, below diagonal) correlations among growth and wood quality traits (standard errors in parentheses).

	DBH	STR	DEN_RES_	DEN_VOL_	VEL_HIT_	VEL_FAK_	VEL_MTG_	MOE_TREE_	MOE_LOG_	MOE_BOARD_
**DBH**		0.30 (0.24)	0.24 (0.24)	−0.39 (0.23)	0.14 (0.34)	−0.13 (0.29)	−0.30 (0.27)	0.21 (0.26)	0.06 (0.28)	**−0.49** (0.23)
**STR**	0.14 (0.03)		0.12 (0.24)	0.08 (0.26)	0.12 (0.33)	0.22 (0.28)	**0.59** (0.21)	0.12 (0.26)	0.33 (0.24)	**0.61** (0.21)
**DEN_RES_**	0.09 (0.03)	0.10 (0.03)		**0.75** (0.12)	**0.78** (0.20)	0.40 (0.24)	0.04 (0.29)	**0.98**^†^ (0.02)	**0.89**^†^ (0.07)	0.46 (0.25)
**DEN_VOL_**	−0.24 (0.04)	0.09 (0.04)	0.72 (0.02)		0.37 (0.31)	0.07 (0.29)	−0.35 (0.28)	**0.69** (0.15)	**0.59** (0.18)	0.21 ^†^ (0.29)
**VEL_HIT_**	0.10 (0.03)	0.15 (0.02)	0.34 (0.02)	0.29 (0.03)		**0.94** (0.17)	**0.74** (0.25)	**0.88**^†^ (0.10)	**0.95** (0.15)	**0.90** (0.21)
**VEL_FAK_**	−0.19 (0.05)	0.09 (0.04)	0.33 (0.04)	0.28 (0.04)	0.43 (0.04)		**0.76** (0.15)	**0.65** (0.18)	**0.71**^†^ (0.15)	**0.86** (0.12)
**VEL_MTG_**	−0.65 (0.03)	0.17 (0.05)	0.25 (0.05)	0.21 (0.05)	0.36 (0.04)	0.60 (0.03)		0.32 (0.26)	0.39 (0.26)	**0.85**^†^ (0.09)
**MOE_TREE_**	0.10 (0.03)	0.15 (0.03)	0.76 ^†^ (0.01)	0.58 (0.03)	0.86 ^†^ (0.01)	0.49 (0.04)	0.39 (0.04)		**0.97**^†^ (0.05)	**0.67** (0.19)
**MOE_LOG_**	−0.10 (0.04)	0.10 (0.04)	0.69 ^†^ (0.02)	0.54 (0.03)	0.40 (0.03)	0.85 ^†^ (0.01)	0.50 (0.04)	0.66 ^†^ (0.02)		**0.75** (0.16)
**MOE_BOARD_**	−0.64 (0.03)	0.18 (0.04)	0.47 (0.04)	0.55 ^†^ (0.03)	0.41 (0.03)	0.61 (0.03)	0.92 ^†^ (0.01)	0.55 (0.03)	0.62 (0.03)	

Note: Genetic correlations with magnitudes greater than two times their estimation error are highlighted in bold. All phenotypic correlations were significant at the 5% level. ^†^ Correlation estimates may be overestimated due to autocorrelation.

**Table 6:** Correlated genetic response, expressed as percentages of the mean, of sawn-board traits to selection based on traits non-destructively measured on standing trees (1% selection intensity).

Selection Traits	Target Traits				
	DBH	DEN_VOL_	MOE_S.local_	MOE_S.global_	MOR
**DBH**	15.19	−1.84	−3.39	−3.33	−0.38
**STR**	4.84	0.38	7.16	4.32	6.90
**DEN_RES_**	4.72	4.51	7.75	6.71	12.97
**VEL_HIT_**	1.00	0.80	4.40	3.13	5.28
**MOE_TREE_**	3.06	3.07	6.69	5.25	10.06

## Appendix A

**Table A1:** Means and standard deviations (*SD*) for traits measured on standing trees selected and unselected for the sawmill study.

Trait	Selected Trees		Unselected Trees		Difference between Means [%]
	*Mean*	*SD*	*Mean*	*SD*	
DBH	19.41	2.79	16.47	4.23	17.9
STR	7.89	0.80	7.82	0.78	0.8
DEN_RES_	526.34	52.17	519.98	53.33	1.2
VEL_HIT_	4.25	0.28	4.21	0.28	0.9
MOE_TREE_	9.55	1.67	9.30	1.82	2.6
